# Infectivity and transmissibility of H9N2 avian influenza virus in chickens and wild terrestrial birds

**DOI:** 10.1186/1297-9716-44-100

**Published:** 2013-10-17

**Authors:** Munir Iqbal, Tahir Yaqub, Nadia Mukhtar, Muhammad Z Shabbir, John W McCauley

**Affiliations:** 1 Avian Viral Diseases Programme, The Pirbright Institute, Compton Laboratory, Compton, Newbury, Berkshire RG20 7NN, UK; 2 Quality Operations Laboratory, University of Veterinary and Animal Sciences, Lahore, Pakistan; 3 Division of Virology, MRC National Institute for Medical Research, Mill Hill, London NW7 1AA, UK

## Abstract

Genetic changes in avian influenza viruses influence their infectivity, virulence and transmission. Recently we identified a novel genotype of H9N2 viruses in widespread circulation in poultry in Pakistan that contained polymerases (PB2, PB1 and PA) and non-structural (NS) gene segments identical to highly pathogenic H7N3 viruses. Here, we investigated the potential of these viruses to cause disease and assessed the transmission capability of the virus within and between poultry and wild terrestrial avian species. Groups of broilers, layers, jungle fowl, quail, sparrows or crows were infected with a representative strain (A/chicken/UDL-01/08) of this H9N2 virus and then mixed with naïve birds of the same breed or species, or different species to examine transmission. With the exception of crows, all directly inoculated and contact birds showed clinical signs, varying in severity with quail showing the most pronounced clinical signs. Virus shedding was detected in all infected birds, with quail showing the greatest levels of virus secretion, but only very low levels of virus were found in directly infected crow samples. Efficient virus intra-species transmission was observed within each group with the exception of crows in which no evidence of transmission was seen. Interspecies transmission was examined between chickens and sparrows and *vice versa* and efficient transmission was seen in either direction. These results highlight the ease of spread of this group of H9N2 viruses between domesticated poultry and sparrows and show that sparrows need to be considered as a high risk species for transmitting H9N2 viruses between premises.

## Introduction

There are seventeen antigenically distinct haemagglutinin (HA) and ten neuraminidase (NA) subtypes of influenza A viruses [1,2]. With the exception of the recently described influenza A (H17N10) virus of bats all other subtypes circulate in birds. Among these, viruses of H5 and H7 are the primary cause of highly pathogenic avian influenza (HPAI) but other sub-types of virus can also cause significant losses in poultry. Low pathogenicity avian influenza (LPAI) viruses of the H9N2 subtype are particularly noteworthy due to their widespread circulation in domestic poultry ranging from the Far East to the Middle East [3-7] and, like HPAI viruses and the new H7N9 influenza virus, pose a significant zoonotic threat [8-13]. Infections with H9N2 viruses have been reported in a diverse variety of wild avian species including quail, crows and sparrows [6,14-18]. Genetic analysis of recent H9N2 viruses has indicated that these H9N2 viruses have undergone extensive reassortment with many subtypes of AI viruses including HPAI H5N1 and H7N3 viruses [4,7,19-21]. These phylogenetic analysis studies suggest that newly emerged reassortants might be maintained in a diverse variety of host species in wide geographical regions [15]. Recent outbreaks of H9N2 virus in poultry in Pakistan show more severe clinical disease signs than previously, unreferenced observations from field veterinarians. In laboratory studies with viruses that were prevalent in 1997 to 2002, which were very closely related in all eight gene segments (97-99% homology) with A/Quail/Hong Kong/G1/97 (G1-lineage) virus, very few disease signs were observed, although some older viruses of the Beijing-lineage (A/Chicken/Beijing/1/94) caused marked disease in experimental studies [6,22]. Recent laboratories studies have also demonstrated that H9N2 viruses belonging to G1-linage is able to cause severe disease in chickens after genetic reassortment by acquiring internal genes from the concurrently circulating HPAI H5N1 virus [23].

The continued circulation of novel reassortant H9N2 viruses depends on many factors but the efficiency of transmission within and between susceptible species is key. A number of studies describe the infectivity and transmissibility of different LPAI and HPAI viruses in different poultry and related land-based wild bird species [6,24-26] but marked variability in infectivity and transmissibility has been seen between different strains within a subtype [6,27] and data obtained from one virus subtype cannot be extrapolated to other virus subtypes or strains.

To date, limited data are available that describe the relative infectivity and transmissibility of H9N2 viruses in different poultry breeds and terrestrial wild bird species. Here, we examined the infection and transmission of a novel genotype of H9N2 virus that was first detected in 2005 and representative of viruses now circulating in poultry in Pakistan and neighbouring Iran, Afghanistan and other regions of Middle East [4,19,21]. This virus genotype acquired PB2, PB1, PA and NS gene segments of H7N3 HPAI virus with the other genes originating in the G1-like lineage of H9N2 viruses [19]. The virus A/chicken/Pakistan/UDL-01/08 (UDL-01/08) representing this genotype was used to assess the infection and transmission between birds within a species (*Gallus gallus*, broilers, layers, and jungle fowl; *Coturnix coturnix*, the common quail; *Passer domesticus*, the house sparrow; and *Corvus splendens*, the house crow) and between species, from infected broilers to naïve sparrows and *vice versa*. The choice was made to represent the breeds of poultry reared in commercial and backyard flocks, and wild-bird species that routinely intermingle with poultry in the Middle East and parts of the Indian sub-continent.

## Materials and methods

### Virus inoculum stocks

The UDL-01/08 H9N2 virus was a field isolate made in University of Veterinary and Animal Sciences, Lahore, Pakistan. The intravenous pathogenicity index (0.00), antigenic and genetic properties (accession no. CY038455 to CY038462) of this virus have been reported [19]. Virus stocks used as inocula were prepared from second passage in 10-day-old embryonating hens’ eggs (EHE). Infective allantoic fluid from the inoculated eggs was diluted in brain-heart infusion buffer (BHIB) and the median egg infectious doses (EID_50_) were determined by the Reed and Muench method [28].

### Animals

Three-week-old white leghorn (layer hens, commercial name LSL), broiler chickens (commercial name Hubbard), red jungle fowl (hen pullets) and common quail (*Coturnix coturnix*) were acquired from a local supplier. House sparrows (*Passer domesticus*) and house crows (*Corvus splendens*) were captured from the wild in the surrounding areas of Lahore, Pakistan. Blood, and buccal and cloacal swab samples were taken from all birds and analysed by haemagglutination inhibition (HI) and virus isolation (VI) in 9-day-old EHE using standard methods [29] to ensure that the birds were serologically naïve and free from influenza virus infection prior to the start of the experiment. Each group of birds was housed separately in cages in separate rooms, food and water were provided *ad libitum*, and general animal care was provided by the animal house staff as required for each species.

### Experimental design

In each experiment, each species of birds were divided into two groups, an infected group and a contact group. For experiments in which transmission within a species or bread was investigated, each infected group contained ten birds and each contact group contained five birds, housed in cages of 2.23 m^2^ floor area. For transmission studies between birds of different species, both infected and contact groups contained ten birds in each group. An uninfected group serving as a negative control group contained five birds of each species. Each bird in the infected group was inoculated intra-nasally with 0.2 mL of UDL-01/08 virus inoculum containing 10^6.6^ EID_50_ and after four hours the uninfected contact birds were mixed with the virus inoculated birds in the same cage. The birds were monitored three times daily for clinical signs (reluctance to move, anorexia, congestion of eyes, respiratory signs mainly sneezing, swollen head, haemorrhage on shanks) and buccal and cloacal swabs were taken at regular intervals on 2–7, 9, 11, 15, 19, 23, 27 and 29 days post infection (dpi). Each experiment lasted for a maximum of four weeks and all surviving birds at end of the experiment were humanely killed.

### Processing of swab samples

Buccal and cloacal swabs collected in 1 mL of 15% BHIB with antibiotics (10 000 IU/mL Pencicillin G + 100 μg/mL Gentamycin + 20 μg/mL Amphotericin B) were kept on ice, and then filtered through a 0.2 μm filter. The filtered material was divided into two aliquots and stored at -80°C until virus isolation.

### Virus isolation and determination of HA titres

Virus isolation was performed by inoculating swab samples into 9-day-old EHE and the presence of virus in the allantoic fluids was determined by virus haemagglutination activity using standard procedures [29].

### Collection of serum samples and HI tests

Blood samples from all birds were collected one day before infection (pre-infection) and on 7, 14, 21, 28 dpi for determination of HI antibody titres against H9N2 virus challenge using the standard HI test [29].

### Statistical analysis

Statistical analysis and graphical presentation was performed using GraphPad Prism 6 software. H9N2-specific antibodies titres in serum were calculated as geometric means and viral titres in buccal and claocal swabs were calculated as medians with standard deviations. Statistical significance (*P* values) between the groups were compared using one-way analysis of variance (ANOVA) and Turkey’s multiple comparison test.

## Results

The analysis of pre-inoculation swab and serum samples by virus isolation and HI tests revealed that all birds used in this study had no detectable infection with H9, H7 or H5 viruses and the levels of HI antibody titres were < 2 Log_2_ against these virus subtypes. The uninfected control groups remained healthy throughout the experiment and showed no clinical disease signs. H9N2-specific HI titres remained at baseline values (≤ 2 Log_2_) in the serum samples recovered from this control group just before termination of the experiments.

### Infection of birds with UDL-01/08

Clinical signs and the susceptibility and transmission of H9N2 virus from infected to naïve contact birds in different species was examined following the inoculation of groups of ten birds (broiler chickens, layer hens, jungle fowl, quail, sparrows and crows) with 10^6.6^ EID_50_ of UDL-01/08. After four hours, the infected birds were co-housed with an additional five naïve uninfected birds of same species in the same cage.

### Clinical signs and duration of virus shedding

Considerable variation in clinical disease signs (from apparent to mild, including general depression, sneezing, respiratory sounds, ocular and nasal discharge, eye redness, ruffled feathers, swollen head and reluctance to move) were observed depending on breed of chickens and wild bird species infected directly with UDL-01/08 or by contact infection from infected birds. The number of birds in each group that showed clinical disease signs is summarised in Table 1. Buccal and cloacal swabs taken from 2–29 dpi were analysed for presence of virus by inoculating into EHE followed by detection of virus haemagglutination activity in the harvested allantoic fluid. The data presented in Table 2 represent summary of the detection of virus in infected birds and in contact birds during the course of infection in broilers, layers, jungle fowls, quail, sparrows and crows. Integration of virus shedding and disease signs on a bird-by-bird basis is shown in Additional file 1, Additional file 2, Additional file 3, Additional file 4, Additional file 5, Additional file 6 and an analysis of the levels of virus in the swab samples is presented below.

**Table 1:** Number of infected and contact birds showing clinical disease signs

**Species**	**Broiler chickens**		**Layers**		**Jungle fowl**		**Quail**		**Sparrows**		**Crows**	
**Clinical signs**	**Infected (10)**	**Contact (5)**	**Infected (10)**	**Contact (5)**	**Infected (10)**	**Contact (5)**	**Infected (10)**	**Contact (5)**	**Infected (10)**	**Contact (5)**	**Infected (10)**	**Contact (5)**
General sickness/	8	5	10	5	8	5	10	5	9	5	-	-
Sneezing	8	1	8	4	8	2	7	4	10	-	-	-
Respiratory sound (rales)	6	1	8	4	-	-x	7	2	10	2	-	-
Occular/nasal discharge	8	1	6	4	-	-	-	-	10	2	-	-
Eye redness	8	2	8	3	-	-	-	-	10	2	-	-
Head Swollen	6	2	7	4	-	-	6	2	6	3	-	-
Ruffled Feathers	6	2	10	5	8	2	10	2	10	-	2	-
Reluctant to move	6	2	4	4	-	-	-	-	6	2	-	-

Infected birds were inoculated with 10^6.6^ EID_50_ UDL-01/08 virus. Clinical signs were observed between 2–5 dpi. The number in parentheses is the total number of birds in a group. (-), indicates no apparent clinical disease signs.

**Table 2:** Number of infected and contact uninfected naïve birds showing the presence of virus in buccal and cloacal swabs collected over 29 days

**Species**	**Infection route**	**Number of birds**	**Passage number**	**Swab samples**	**Days post infection**												
					**2**	**3**	**4**	**5**	**6**	**7**	**9**	**11**	**15**	**19**	**23**	**27**	**29**
Broilers	Infected	10	1^st^	B	5	10	10	10	10	10	10	9	2	-	-	-	-
				C	-	10	10	10	4	4	-	-	-	-	-	-	-
	**Contact**	**5**	**1**^**st**^	**B**	**2**	**3**	**3**	**4**	**4**	**4**	**4**	**4**	**-**	**-**	**-**	**-**	**-**
				**C**	**-**	**5**	**5**	**5**	**1**	**-**	**-**	**-**	**-**	**-**	**-**	**-**	**-**
Layers	Infected	10	1^st^	B	7	10	10	10	10	10	10	10	-	-	-	-	-
				C	-	10	10	10	-	-	-	-	-				
	**Contact**	**5**	**1**^**st**^	**B**	**1**	**4**	**3**	**4**	**4**	**4**	**4**	**2**	**-**	**-**	**-**	**-**	**-**
				**C**	**-**	**5**	**5**	**4**	**2**	**-**	**-**	**-**	**-**	**-**	**-**	**-**	**-**
Jungle fowl	Infected	10	1^st^	B	7	10	10	10	10	10	10	10	1	- -	- -	- -	- --
				C	-	10	10	10	6	3	-	-	-				
	**Contact**	**5**	**1**^**st**^	**B**	**3**	**2**	**2**	**2**	**2**	**2**	**1**	**1**	**-**	**-** **-**	**-** **-**	**-** **-**	**-** **-**
				C	-	5	5	5	4	-	-	-	-				
Quail	Infected	10*	1^st^	B	10	10	8	8	8	8	6	-	-	- -	- -	- -	- -
				C	-	10	8	8	7	1	-	-	-				
	**Contact**	**5**	**1**^**st**^	**B**	**4**	**5**	**5**	**4**	**4**	**2**	**-**	**-**	**-** **-**	**-** **-**	**-** **-**	**-** **-**	**-** **-**
				**C**	**-**	**5**	**5**	**5**	**5**	**2**	**-**	**-**					
Sparrows	Infected	10	1^st^	B	10	10	10	10	10	10	3	-	-	-	-	-	-
				C	-	10	10	10	6	-	-	-	-	-	-	-	-
	**Contact**	**5**	**1**^**st**^	**B**	**5**	**5**	**4**	**4**	**4**	**3**	**-**	**-**	**-**	**-**	**-**	**-**	**-**
				**C**	**-**	**5**	**5**	**5**	**4**	**-**	**-**	**-**	**-**	**-**	**-**	**-**	**-**
Crows*	Infected	10	1^st^	B	-	-	-	-	-	-	-	-	-	-	-	-	-
				C	-	-	-	-	-	-	-	-	-	-	-	-	-
			2^nd^	B	1	9	9	10	10	8	-	-	-	-	-	-	-
				C	-	10	10	10	6	1	-	-	-	-	-	-	-
	**Contact**	**5**	**1**^**st**^	**B**	**-**	**-**	**-**	**-**	**-**	**-**	**-**	**-**	**-**	**-**	**-**	**-**	**-**
				**C**	**-**	**-**	**-**	**-**	**-**	**-**	**-**	**-**	**-**	**-**	**-**	**-**	**-**
			**2**^**nd**^	**B**	**-**	**-**	**-**	**-**	**-**	**-**	**-**	**-**	**-**	**-**	**-**	**-**	**-**
				**C**	**-**	**-**	**-**	**-**	**-**	**-**	**-**	**-**	**-**	**-**	**-**	**-**	**-**

*swabs from crows only showed virus presence after two serial passages in eggs. B, denotes buccal swabs. C, denotes cloacal swabs and -, indicates that swabs were negative for virus. Infected birds were inoculated with 10^6.6^ EID_50_ UDL-01/08 virus. Swab samples were inoculated into EHE and virus in the harvested allantoic fluid was detected by HA assay. * two quail on 4 dpi died and from this time point onward swabs from 8 birds were analysed. Contact birds are indicated in bold.

All (10/10) of the broilers and layers were successfully infected and the presence of virus both the buccal cavity and cloaca was from 3 dpi which resolved by days 11 or 15. Only eight of the ten broilers showed clinical signs; these varied in severity and the signs that were apparent were only observed on days 3 to 5. All layers also showed clinical signs, with the disease signs observed between days 1 to 5, but with the majority of signs being seen, like in the broilers, on days 2 to 5 (Additional file 1 and Additional file 2). All experimentally challenged jungle fowl showed presence of virus within 3 dpi and eight of the ten infected birds displayed mild clinical disease signs limited to sneezing and ruffled feathers but of generally shorter duration than the signs seen in the chickens, and all signs had regressed by day 5. Virus shedding in jungle fowl was not detected after 11 days (Additional file 3). All 10 directly inoculated quail showed presence of virus in buccal cavity from 2 dpi with continued until day 9 and all showed severe disease signs (sneezing, rales, head swollen and ruffled feathers) from 3–5 dpi. Two infected quail died at 4 dpi, possibly from infection but no post-mortem was performed. Once again no disease signs were seen after day 5 post infection (Additional file 4). As with the other species, all directly infected sparrows (10/10) were positive for virus infection, shedding virus from day 2 to days 7 or 9; they showed with pronounced clinical signs (sneezing, respiratory sounds, ocular and nasal discharge, eye redness) observed between 3 and 5 dpi (Additional file 5). All (10/10) crows showed presence of virus in buccal cavity and cloaca after 2 dpi but only after a second passage in eggs, all shedding had ceased by day 9 after infection. Two of the ten infected crows showed slightly ruffled feathers with no other apparent clinical disease signs (Additional file 6). It is striking that virus shedding continued after any disease signs had disappeared in all species.

Generally the contact groups showed similar signs of infection and duration of shedding to the directly inoculated birds with the exception of contact crows. Infection was typically delayed by a day and the duration of shedding was similar. There was no evidence that contact crows were infected when housed with the directly infected crows, the considerably lowered levels of virus being detected in the buccal and cloacal cavities probably being a factor in the inefficient spread of the virus.

Analysis of cloacal swab samples taken from infected birds showed that the presence of virus in the cloacal cavity was delayed for one day compared with the buccal cavity.

These results indicated that broilers, layers, Jungle fowl, quail and sparrows were highly susceptible to UDL-01/08 H9N2 virus infection and able to transmit virus to uninfected birds of same species efficiently, when kept together within same cage. The crows showed a low level of susceptibility to virus with the initial passage of swab samples in eggs proving negative as assessed by HA, on additional passage of the allantoic fluid virus was detected.

### Transmission between broilers and sparrows

In light of the results of interspecies transmission of UDL-01/08 H9N2 virus its transmission from infected chickens to naïve sparrows was assessed. A group of broilers was inoculated with UDL-01/08 and after 4 h, a group of naïve sparrows was mixed with the infected broilers in the same cage. Analysis of buccal and cloacal swabs taken at different time points from 2–28 dpi from each infected and contact bird revealed that all inoculated birds were infected and all of the contact sparrows became infected; the infected broilers showed virus shedding up to 14 dpi and the contact sparrows up to 10 dpi (Table 3). The comparison of virus shedding data from chickens and sparrows revealed that all infected birds had virus in buccal and cloacal cavities from 3–8 dpi. The contact sparrows were all infected by 4 dpi, and all shed virus from cloaca until 8 dpi. Infection of the sparrows had resolved by 14 dpi (Table 3).

**Table 3:** Virus isolation from buccal and cloacal swabs collected over 28 days from infected broilersand contact naïve sparrow

	**Species**	**Number of birds**	**Swab samples**	**Days post infection**								
				**2**	**3**	**4**	**6**	**8**	**10**	**14**	**21**	**28**
Cage 1	Infected broilers	10	B	4	10	10	10	10	6	2	-	-
			C	-	10	10	10	10	6	1	-	-
	**Contact sparrows**	**10**	**B**	**2**	**5**	**10**	**6**	**1**	**3**	**-**	**-**	**-**
			**C**	**-**	**-**	**10**	**10**	**10**	**7**	**-**	**-**	**-**
Cage 2	Infected sparrows	10	B	10	10	10	10	10	1	-	-	-
			C	8	10	10	10	10	8	3	-	-
	**Contact broilers**	**10**	**B**	**-**	**2**	**10**	**10**	**10**	**8**	**-**	**-**	**-**
			**C**	**-**	**-**	**6**	**10**	**10**	**10**	**1**	**-**	**-**

B, denotes buccal swabs. C, denotes cloacal swabs and -, indicated that the swabs showed no virus presence. Infected birds were inoculated with 10^6.6^ EID_50_ UDL-01/08 virus. Swab samples were inoculated into EHE and virus was detected by HA assay. Contact birds are indicated in bold.

Next to answer the reciprocal question whether infected sparrows can transmit virus to naïve broilers, a group of sparrows was inoculated with UDL-01/08 and, after 4 h, a group of naïve broilers was mixed with infected sparrows. Analysis of buccal and cloacal swabs again taken at different time points from 2–28 dpi from each bird revealed that the buccal and cloacal swabs taken from inoculated sparrows and contact broilers were positive for virus. All inoculated (10/10) sparrows had virus in their buccal and cloacal cavities from 3–8 dpi and all contact broilers showed presence of virus in both buccal cavity and cloaca on 6–8 dpi. These results indicate that virus can pass from sparrows to broilers and *vice versa* (Table 3).

### Viral titres in buccal and cloacal swabs in infected and contact birds

**Figure 1:**
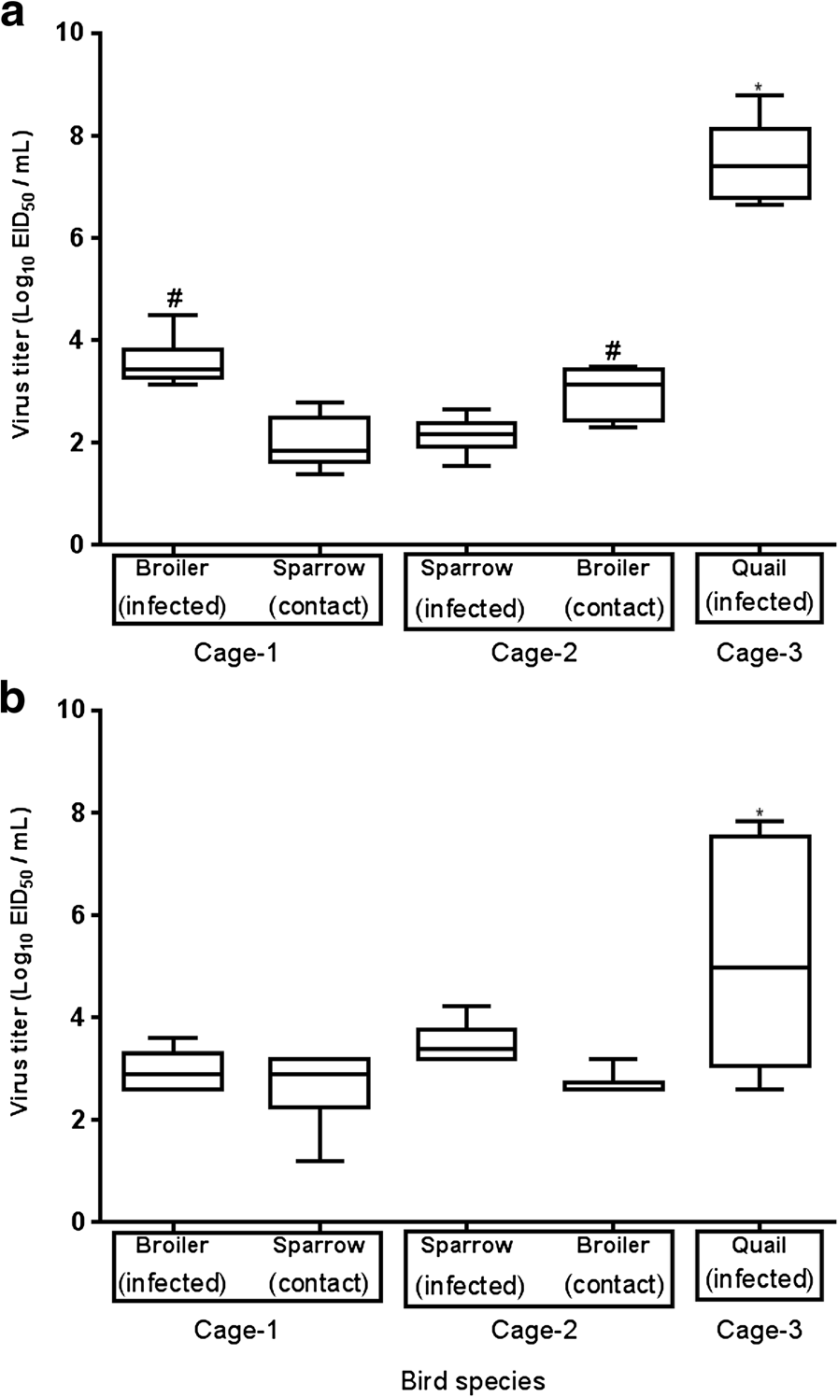
**Viral titres (EID**_**50 **_**/mL) determined in EHE inoculated with (a) buccal and (b) cloacal swabs taken from the infected groups on 3 dpi and from the contact groups on 4 dpi.** Infected broilers and naïve contact sparrows were housed together in cage 1, infected sparrows and naïve contact broilers were housed together in cage 2 and infected quail were housed in cage 3. The square below the axis represents the cages. Data are presented as box-and-whisker plot indicating maximum and minimum titres observed in six birds selected from each group that were positive for virus infection within the infected and contact groups. The horizontal line marks the median and the error bars indicate standard deviation. (*) indicates significant differences (*P* < 0.05) compared with both of the broiler and sparrow groups and (#) indicates significant differences (*P* < 0.05) compared with two sparrow groups. Differences were calculated using ANOVA and Tukey’s multiple comparison tests.

In order to determine levels of virus replication in the respiratory and gastrointestinal tract in infected broilers and contact sparrows, infected sparrows and contact broilers, and infected quail, six virus positive buccal and cloacal swabs taken on 3 dpi from infected birds and 4 dpi from contact birds were titrated in EHE. Median viral titres were 10^3.5^ EID_50_/mL (range, 10^3.1^ to 10^4.5^ EID_50_/mL) in buccal swabs of infected broilers and 10^1.9^ EID_50_/mL (range, 10^1.4^ to 10^2.8^ EID_50_/mL) in contact sparrows. Median viral titres in buccal swabs were 10^2.2^ EID_50_/mL (range, 10^1.5^ to 10^2.6^ EID_50_/mL) on 3 dpi in infected sparrows and 10^3.2^ EID_50_/mL (range, 10^2.3^ to 10^3.5^ EID_50_/mL) on 4 dpi in contact broilers (Figure 1a). The cloacal swabs taken from the infected broilers on 3 dpi and from contact sparrows on 4 dpi contained median viral titres 10^2.9^ EID_50_/mL (range, 10^2.6^ to 10^3.6^ and 10^1.2^ to 10^3.2^, respectively). The cloacal swabs recovered from the infected sparrow group and the contact broiler group contained median viral titres 10^3.4^ EID_50_/mL (range, 10^3.2^ to 10^4.2^ EID_50_/mL) and 10^2.6^ (range, 10^2.6^ to 10^3.2^ EID_50_/mL), respectively (Figure 1b). The buccal and cloacal swabs taken from infected quail contained median virus titres 10^7.4^ EID_50_/mL (range, 10^6.6^ to 10^8.7^ EID_50_/mL) and 10^5.0^ EID_50_/mL (range, 10^2.6^ to 10^7.8^ EID_50_/mL), respectively, which were significantly greater (*p* < 0.05) than the viral titres observed in buccal and cloacal swabs taken from broilers and sparrows (Figure 1). Mean viral titers in buccal and cloacal swabs taken from the infected and contact groups are presented in Additional file 7. These data demonstrated that sparrows shed significantly less (*P* < 0.05) virus from oral route compared with broilers and quail but there were no significant differences (*P* > 0.05) in cloacal shedding between infected sparrows and broilers; thereby efficient virus transmission was possible between infected sparrows and contact chickens.

### Serum antibody titres in infected and contact birds

Detection of virus-specific antibodies in infected animals was examined as evidence for the establishment of infection. Serum samples taken from both the directly inoculated groups and the contact groups at one day before infection (pre-infection samples), 7 dpi, 14 dpi, 21 dpi and 28 dpi were analysed by HI assays (Table 4). In infected groups, on 7 dpi, all (10/10) broilers, layers and sparrows, 9/10 jungle fowl and 8/8 quail were positive for H9N2 virus–specific antibodies. With the exception of the crow group, all infected groups showed the presence of H9N2 virus-specific antibodies from 14–28 dpi with 8.0 and 9.7 log_2_ HI geometric mean titre (GMT). Infected crows were positive for HI antibodies from 21–28 dpi with of 6.9 – 7.7 GMT. In contact groups, broilers showed the presence of antibody from 7–28 dpi and layers, jungle fowl, quail and sparrows contained positive HI titres from 14–28 dpi. No antibodies against H9N2 virus were detected in the contact crow group, suggesting that crows are relatively resistant to H9N2 infection and inter-host transmission of H9N2 viruses in crows is inefficient compared with that observed in chickens, quail and sparrows.

**Table 4:** HI titres of sera collected from UDL-01/08 virus infected and contact birds

**Species**	**Infection route**	**Pre-infection HI titre**	**7 dpi**	**14 dpi**	**Day 21 dpi**	**28 dpi**
			**Number of birds positive for HI / total birds**			
			**log**_**2 **_**HI titer range (GMT)**			
Broiler	Infected	<2	10/10	10/10	10/10	10/10
			5–8 (6.2)	7–9 (8.0)	8–9 (8.4)	8–9 (8.4)
	**Contact**	**<2**	**5/5**	**5/5**	**5/5**	**5/5**
			**3–6 (4.1)**	**5–7 (5.7)**	**8–9 (8.3)**	**8–10 (8.9)**
Layer	Infected	<2	10/10	10/10	10/10	10/10
			5–8 (6.1)	7–9 (8.0)	8–9 (8.4)	8–9 (8.6)
	**Contact**	**<2**	**0/5**	**5/5**	**5/5**	**5/5**
			**<2**	**5–7 (6.0)**	**8–9 (8.4)**	**8–9 (8.4)**
Jungle fowl	Infected	<2	9/10	10/10	10/10	10/10
			7–9 (7.5)	9–10 (9.1)	9–10 (9.6)	9–10 (9.6)
	**Contact**	**<2**	**0/5**	**5/5**	**5/5**	**5/5**
			**<2**	**6–8 (7.4)**	**9–10 (9.6)**	**9–10 (9.6)**
Quail	Infected	<2	8/8	8/8	8/8	8/8
			6–8 (7.5)	7–10 (8.6)	8–10 (8.9)	9–10 (9.4)
	**Contact**	**<2**	**0/5**	**5/5**	**5/5**	**5/5**
			**<2**	**7–9 (7.8)**	**8–9 (8.6)**	**8–9 (8.6)**
Sparrow	Infected	<2	10/10	10/10	10/10	10/10
			5–8 (6.5)	7–9 (8.2)	8–10 (8.8)	9–10 (9.7)
	**Contact**	**<2**	**0/5**	**5/5**	**5/5**	**5/5**
			**<2**	**7–9 (7.6)**	**8–10 (8.8)**	**8–9 (8.1)**
Crow	Infected	<2	0/10	0/10	10/10	10/10
			<2	<2	4–9 (6.9)	5–9 (7.7)
	**Contact**	**<2**	**0/5**	**0/5**	**0/5**	**0/5**
			**<2**	**<2**	**<2**	**<2**

GMT, Geometric mean titers; dpi, days post infection. HI titres <2 were regarded as baseline detection and were considered negative for seroconversion. Infected birds were inoculated with 10^6.6^ EID_50_ UDL-01/08 virus. Swab samples were inoculated into EHE and virus was detected by HA assay. Contact birds are indicated in bold.

## Discussion

A novel genotype of H9N2 viruses containing HA, NA, NP and M gene segments similar to A/quail/Hong Kong/G1/97, but the polymerase (PB2, PB1, PA) and non-structural (NS) gene segments identical to H7N3 virus, is prevalent in poultry in regions of the Indian subcontinent and the Middle East [4,19,21]. The infectivity of a representative virus of this genotype (UDL-01/08) to a variety of species was assessed and transmission from infected to contact birds was examined. Infection with UDL-01/08 virus induced clinical disease signs in broilers, layers, jungle fowl, quail and sparrow, but infected crows remained healthy and showed no clinical disease signs. The results of this study support field data in which broilers and layers infected with currently prevalent H9N2 viruses show increased clinical disease compared with infection with H9N2 viruses that were causing outbreaks in chickens in 1999 ([6] and unreferenced experience from field veterinarians). These older viruses were genetically more closely related to A/quail/Hong Kong G1/97 in all eight gene segments [22] and are genetically distinct from the currently circulating reassortant virus [19]. Experimental infection of chickens with A/quail/Hong Kong G1/97 has been reported to induce no clinical disease signs following experimental infection [6], which was reproduced in our unpublished results. Similar observations have been made for other strains of H9N2 viruses [23,30,31]; these showed that, although quail are largely susceptible to H9N2 viruses, infection of quail and chickens with a range of H9N2 viruses isolated from ducks, chickens or quail did not induce clinical disease. Other studies that examined experimental infection of quail with H9N2 viruses of the G1 lineage (A/chicken/Iran/ZMT-101/98, A/chicken/Iran/SH-110/99 and A/Chicken/Iran/339/02) prevalent in poultry in Iran also showed pronounced clinical disease signs [32-34]. Recent studies have also demonstrated that a laboratory generated reassortant H9N2 virus carrying the internal genes of HPAI H5N1 can cause severe disease in chickens [23]. These results are similar to our observations with the new reassortant UDL-01/08, which induced marked clinical disease signs not only in quail but also in chickens. Notably, two infected quail became severely ill due to infection and died 4 dpi. It is striking that house sparrows were highly susceptible to UDL-01/08 virus and showed clinical disease signs but other studies with H9N2 infections of sparrows have not, to our knowledge, been reported.

Under our experimental conditions, it is highly likely that chickens, quail and sparrows showed clinical disease signs directly due to H9N2 virus infection and not with secondary infections. Importantly, the birds in the control uninfected group remained healthy throughout the experiment and showed no apparent clinical disease signs. Nevertheless, we have not undertaken tests to screen these birds for any subclinical disease. Previously, it was widely believed that H9N2 infections are not the main cause of the induction of disease signs in chickens, but instead farm conditions (together with bacterial or viral co-infections, age and breed of chickens) were important contributing factors for higher morbidity and mortality [35-37]. Like ours, other experimental studies involving H9N2 infections in commercial broiler chickens showed clinical disease signs [35,38]. Nevertheless, the concurrent infections of other viral and bacterial pathogens have been shown to exacerbate the clinical outcome of H9N2 infection in chickens [38-40].

Our data indicate that sparrows are highly susceptible to H9N2 infection and infected sparrows can transmit virus not only to contact sparrows but also to contact chickens. However, crows were relatively resistant to H9N2 virus infection and showed only very low levels of virus multiplication resulting in a low amount of virus being shed and no virus transmission to contact crows. Our studies did not quantify very low levels of virus RNA in the swabs by real-time PCR which was not used in the analyses; moreover, the quantitative results for virus infectivity shown in Figure 1 could not be applied to the low titres of viable virus propagated from swabs from crows since virus was only detected after a blind passage (Table 2). It is known that crows are susceptible to H5N1 avian influenza virus infection [25,41-50], but, to our knowledge, no experimental infection has been reported describing the levels of virus shedding and transmission from H5N1 infected crows to naïve contact crows or chickens. Evidence for widespread prevalence of H9N2 viruses in house sparrows and crows is only limited [14,16], but six H9N2 viruses collected from sparrows in 2005 and 2006 have been described, and the HA genes have been characterised (Zhu,W. and Dong, J.B., unpublished, Table 5). The gene sequences of these viruses were similar to those isolated at the same time from poultry in Guangxi province of China. This genetic data suggest that there is an interchange of virus between poultry and sparrows in regards to this genotype of H9N2 virus. Similar cross-species transmission seems likely also to be possible, based on our experimental studies, with the new genotype of H9N2 virus in circulation in Pakistan and the Middle-East. Other factors that might influence the impact of the virus in Pakistan and the surrounding area is the increased disease signs observed by field veterinarians, supported by our studies here, or increased virus multiplication in the infected host of the newly emerged genotype.

**Table 5:** Percentage nucleotide similarities between of HA gene of H9N2 viruses isolated from sparrows, chickens and swine

**H9N2 Viruses isolated from Sparrows**	**Closely related H9N2 viruses in chicken, sparrows and pigs**	**Accession no**^**a**^	**% identify**
	A/chicken/Guangxi/44/2006	GU722362	99.5
	A/chicken/Guangxi/21/2006	GU722360	99.4
A/sparrow/Guangxi/31/2006	A/chicken/Guangxi/55/2005	EU086245	99.3
accession no. GU722366	A/sparrow/Guangxi/11/2005	GU722365	99.4
	A/duck/Beijing/31/2005	GQ373068	98.7
	A/chicken/Beijing/7/2005	GQ373083	99.1
	A/sparrow/Guangxi/93/2006	GU722368	99.6
A/sparrow/Guangxi/09/2005	A/chicken/Guangxi/521/2005	CY023728	99.6
accession no. GU722364	A/chicken/Guangxi/2441/2004	CY023704	99.3
	A/swine/Guangxi/58/2005	EF612742	98.0

^a^HA nucleotide sequences were retrieved on January 5, 2013 from GenBank influenza virus database.

In conclusion, our results indicated that reassortant H9N2 viruses currently circulating in the Indian sub-continent and the Middle East can cause clinical disease in experimentally infected commercial and backyard chicken breeds as well as in quail and house sparrows. Our observations of efficient transmission between different chicken breeds, from quail to quail, and from chickens to sparrows or *vice versa* suggest a worrying scenario in which quail and sparrows could be potential intermediate hosts for maintenance of viruses and the transmission of these viruses to poultry. Crows seem less of a problem because, although they became infected, they did not transmit the virus to contacts and had only low virus titres in swabs. In light of these results, there is a need for increased surveillance of circulating H9N2 and other low pathogenicity AIV such as the novel H7N9 viruses detected in poultry, ducks and pigeons in China, a virus with six RNA segments derived from H9N2 viruses [51,52]. Further experimental infection studies of new reassortant viruses with genes from H9N2 viruses will be needed to assess their pathogenicity and transmissibility in different poultry hosts and terrestrial wild bird species, especially those that might act as carriers and as a reservoir of infection and potentially shuttle the viruses between poultry and, possibly, to humans.

## Abbreviations

NS: Non-structural; HA: Haemagglutinin; NA: Neuraminidase; HPAI: Highly pathogenic avian influenza; UDL-01/08: A/chicken/UDL-01/08; EHE: Embryonating hens’ eggs; EID50: 50% egg infectious dose; HI: Haemagglutination inhibition; VI: Virus isolation; dpi: Days post infection; BHIB: Brain heart infusion buffer; GMT: Geometric mean titre.

## Competing interests

The authors declare that they have no competing interests.

## Authors’ contributions

Conceived and designed the experiments: MI, JWM. Performed the experiments: TY, NM, MZS. Analysed the data: MI, TY, NM, MZS, JWM. Wrote the paper: MI, JWM. All authors read and approved the final manuscript.

## Supplementary Material

Additional file 1 **Clinical disease signs and presence of virus in buccal and cloacal swabs collected from infected and contact broilers.** Infected broilers were inoculated with 10^6.6^ EID_50_ UDL-01/08 virus and after four hours the uninfected contact broilers were mixed in the same cage with the virus inoculated broilers. Swab samples were inoculated in EHE and virus in the harvested allantoic fluid was detected by HA assay. Y, denotes presence of clinical disease signs and presence of virus in buccal and cloacal swabs. -, denotes absence of clinical disease signs and virus in buccal and cloacal swabs. Click here for file

Additional file 2 **Clinical disease signs and presence of virus in buccal and cloacal swabs collected from infected and contact layers.** Infected layers were inoculated with 10^6.6^ EID_50_ UDL-01/08 virus and after four hours the uninfected contact layers were mixed in the same cage with the virus inoculated layers. Swab samples were inoculated in EHE and virus in the harvested allantoic fluid was detected by HA assay. Y, denotes presence of clinical disease signs and presence of virus in buccal and cloacal swabs. -, denotes absence of clinical disease signs and virus in buccal and cloacal swabs. Click here for file

Additional file 3 **Clinical disease signs and presence of virus in buccal and cloacal swabs collected from infected and contact Jungle fowl.** Infected Jungle fowl were inoculated with10^6.6^ EID_50_ UDL-01/08 virus and after four hours the uninfected contact Jungle fowl were mixed in the same cage with the virus inoculated Jungle fowl. Swab samples were inoculated in EHE and virus in the harvested allantoic fluid was detected by HA assay. Y, denotes presence of clinical disease signs and presence of virus in buccal and cloacal swabs. -, denotes absence of clinical disease signs and virus in buccal and cloacal swabs. Click here for file

Additional file 4 **Clinical disease signs and presence of virus in buccal and cloacal swabs collected from infected and contact quail.** Infected quail were inoculated with10^6.6^ EID_50_ UDL-01/08 virus and after four hours the uninfected contact quail were mixed in the same cage with the virus inoculated quail. Swab samples were inoculated in EHE and virus in the harvested allantoic fluid was detected by HA assay. Y, denotes presence of clinical disease signs and presence of virus in buccal and cloacal swabs. -, denotes absence of clinical disease signs and virus in buccal and cloacal swabs. Click here for file

Additional file 5 **Clinical disease signs and presence of virus in buccal and cloacal swabs collected from infected and contact sparrows.** Infected sparrows were inoculated with 10^6.6^ EID_50_ UDL-01/08 virus and after four hours the uninfected contact sparrows were mixed in the same cage with the virus inoculated sparrows. Swab samples were inoculated in EHE and virus in the harvested allantoic fluid was detected by HA assay. Y, denotes presence of clinical disease signs and presence of virus in buccal and cloacal swabs. -, denotes absence of clinical disease signs and virus in buccal and cloacal swabs. Click here for file

Additional file 6 **Clinical disease signs and presence of virus in buccal and cloacal swabs collected from infected and contact crows.** Infected crows were inoculated with 10^6.6^ EID_50_ UDL-01/08 virus and after four hours the uninfected contact crows were mixed in the same cage with the virus inoculated crows. Swab samples were inoculated in EHE and virus in the harvested allantoic fluid was detected by HA assay. Y, denotes presence of clinical disease signs and presence of virus in buccal and cloacal swabs. -, denotes absence of clinical disease signs and virus in buccal and cloacal swabs. Click here for file

Additional file 7 **Mean viral titers in buccal and cloacal swabs taken from the infected groups on 3 dpi and from contact groups on 4 dpi.** Viral titres are presented as log_10_ EID_50_/mL determined in EHE inoculated with buccal and cloacal swabs taken from the infected groups on 3 dpi and from the contact groups on 4 dpi. SD, denotes standard deviation. Click here for file
