# Supplementary material for: Infectivity and transmissibility of H9N2 avian influenza virus in chickens and wild terrestrial birds
Source: Vet Res. 2013 Oct 17;44(1):100. doi: 10.1186/1297-9716-44-100 (PMC4015117; doi:10.1186/1297-9716-44-100)
Supplement: Additional file 3 — Clinical disease signs and presence of virus in buccal and cloacal swabs collected from infected and contact Jungle fowl. Infected Jungle fowl were inoculated with106.6 EID50 UDL-01/08 virus and after four hours the uninfected contact Jungle fowl were mixed in the same cage with the virus inoculated Jungle fowl. Swab samples were inoculated in EHE and virus in the harvested allantoic fluid was detected by HA assay. Y, denotes presence of clinical disease signs and presence of virus in buccal and cloacal swabs. -, denotes absence of clinical disease signs and virus in buccal and cloacal swabs. [file 1297-9716-44-100-S3.docx]

| **Jungle fowl** | | | | | | | | | | | | | | | | | | | | | |
| --- | --- | --- | --- | --- | --- | --- | --- | --- | --- | --- | --- | --- | --- | --- | --- | --- | --- | --- | --- | --- | --- |
| **Clinical Sign** | | **Infected** | | | | | | | | | | **Bird ID** | **Contact** | | | | | | | | |
|  |  | **Bird ID** | **Day Post Infection** | | | | | | | | |  | **Day Post Infection** | | | | | | | | |
|  |  |  | **2** | **3** | **4** | **5** | **6** | **7** | **9** | **11** | **15** |  | **2** | **3** | **4** | **5** | **6** | **7** | **9** | **11** | **15** |
| General Sickness | | J1 | - | Y | Y | - | - | - | - | - | - | J11 | - | Y | Y | - | - | - | - | - | - |
| Sneezing | |  | - | Y | Y | - | - | - | - | - | - |  | - | Y | Y | - | - | - | - | - | - |
| Respiratory sound (rales) | |  | - | - | - | - | - | - | - | - | - |  | - | - | - | - | - | - | - | - | - |
| Ocular nasal discharge | |  | - | - | - | - | - | - | - | - | - |  | - | - | - | - | - | - | - | - | - |
| Eye redness | |  | - | - | - | - | - | - | - | - | - |  | - | - | - | - | - | - | - | - | - |
| Head swollen | |  | - | - | - | - | - | - | - | - | - |  | - | - | - | - | - | - | - | - | - |
| Ruffled feathers | |  | - | Y | Y | - | - | - | - | - | - |  | - | Y | Y | - | - | - | - | - | - |
| Reluctant to move | |  | - | - | - | - | - | - | - | - | - |  | - | - | - | - | - | - | - | - | - |
| Shedding | Buccal |  | Y | Y | Y | Y | Y | Y | Y | Y | - |  | Y | Y | Y | Y | Y | Y | Y | Y | - |
|  | Cloacal |  | - | Y | Y | Y | Y | - | - | - | - |  | - | Y | Y | Y | Y | - | - | - | - |
| General Sickness | | J2 | Y | Y | Y | - | - | - | - | - | - | J12 | - | Y | Y | - | - | - | - | - | - |
| Sneezing | |  | - | Y | Y | - | - | - | - | - | - |  | - | - | - | - | - | - | - | - | - |
| Respiratory sound (rales) | |  | - | - | - | - | - | - | - | - | - |  | - | - | - | - | - | - | - | - | - |
| Ocular nasal discharge | |  | - | - | - | - | - | - | - | - | - |  | - | - | - | - | - | - | - | - | - |
| Eye redness | |  | - | - | - | - | - | - | - | - | - |  | - | - | - | - | - | - | - | - | - |
| Head swollen | |  | - | - | - | - | - | - | - | - | - |  | - | - | - | - | - | - | - | - | - |
| Ruffled feathers | |  | - | Y | Y | - | - | - | - | - | - |  | - | - | - | - | - | - | - | - | - |
| Reluctant to move | |  | - | - | - | - | - | - | - | - | - |  | - | - | - | - | - | - | - | - | - |
| Shedding | Buccal |  | Y | Y | Y | Y | Y | Y | Y | Y | - |  | - | - | - | - | - | - | - | - | - |
|  | Cloacal |  | - | Y | Y | Y | - | - | - | - | - |  | - | Y | Y | Y | - | - | - | - | - |
| General Sickness | | J3 | - | - | - | - | - | - | - | - | - | J13 | Y | Y | Y | - | - | - | - | - | - |
| Sneezing | |  | - | - | - | - | - | - | - | - | - |  | - | Y | Y | - | - | - | - | - | - |
| Respiratory sound (rales) | |  | - | - | - | - | - | - | - | - | - |  | - | - | - | - | - | - | - | - | - |
| Ocular nasal discharge | |  | - | - | - | - | - | - | - | - | - |  | - | - | - | - | - | - | - | - | - |
| Eye redness | |  | - | - | - | - | - | - | - | - | - |  | - | - | - | - | - | - | - | - | - |
| Head swollen | |  | - | - | - | - | - | - | - | - | - |  | - | - | - | - | - | - | - | - | - |
| Ruffled feathers | |  | - | - | - | - | - | - | - | - | - |  | - | Y | Y | - | - | - | - | - | - |
| Reluctant to move | |  | - | - | - | - | - | - | - | - | - |  | - | - | - | - | - | - | - | - | - |
| Shedding | Buccal |  | - | Y | Y | Y | Y | Y | Y | Y | - |  | Y | Y | Y | Y | Y | Y | - | - | - |
|  | Cloacal |  | - | Y | Y | Y | - | - | - | - | - |  | - | Y | Y | Y | Y | - | - | - | - |
| General Sickness | | J4 | - | Y | Y | - | - | - | - | - | - | J14 | - | Y | Y | - | - | - | - | - | - |
| Sneezing | |  | - | Y | Y | - | - | - | - | - | - |  | - | - | - | - | - | - | - | - | - |
| Respiratory sound (rales) | |  | - | - | - | - | - | - | - | - | - |  | - | - | - | - | - | - | - | - | - |
| Ocular nasal discharge | |  | - | - | - | - | - | - | - | - | - |  | - | - | - | - | - | - | - | - | - |
| Eye redness | |  | - | - | - | - | - | - | - | - | - |  | - | - | - | - | - | - | - | - | - |
| Head swollen | |  | - | - | - | - | - | - | - | - | - |  | - | - | - | - | - | - | - | - | - |
| Ruffled feathers | |  | - | Y | Y | - | - | - | - | - | - |  | - | - | - | - | - | - | - | - | - |
| Reluctant to move | |  | - | - | - | - | - | - | - | - | - |  | - | - | - | - | - | - | - | - | - |
| Shedding | Buccal |  | Y | Y | Y | Y | Y | Y | Y | Y | - |  | - | - | - | - | - | - | - | - | - |
|  | Cloacal |  | - | Y | Y | Y | Y | Y | - | - | - |  | - | Y | Y | Y | Y | - | - | - | - |
| General Sickness | | J5 | - | Y | Y | - | - | - | - | - | - | J15 | - | Y | Y | - | - | - | - | - | - |
| Sneezing | |  | - | Y | Y | - | - | - | - | - | - |  | - | - | - | - | - | - | - | - | - |
| Respiratory sound (rales) | |  | - | - | - | - | - | - | - | - | - |  | - | - | - | - | - | - | - | - | - |
| Ocular nasal discharge | |  | - | - | - | - | - | - | - | - | - |  | - | - | - | - | - | - | - | - | - |
| Eye redness | |  | - | - | - | - | - | - | - | - | - |  | - | - | - | - | - | - | - | - | - |
| Head swollen | |  | - | - | - | - | - | - | - | - | - |  | - | - | - | - | - | - | - | - | - |
| Ruffled feathers | |  | - | Y | Y | - | - | - | - | - | - |  | - | - | - | - | - | - | - | - | - |
| Reluctant to move | |  | - | - | - | - | - | - | - | - | - |  | - | - | - | - | - | - | - | - | - |
| Shedding | Buccal |  | - | Y | Y | Y | Y | Y | Y | Y | - |  | Y | - | - | - | - | - | - | - | - |
|  | Cloacal |  | - | Y | Y | Y | - | - | - | - | - |  | - | Y | Y | Y | Y | - | - | - | - |
| General Sickness | | J6 | - | Y | Y | - | - | - | - | - | - |  |  |  |  |  |  |  |  |  |  |
| Sneezing | |  | - | Y | Y | - | - | - | - | - | - |  |  |  |  |  |  |  |  |  |  |
| Respiratory sound (rales) | |  | - | - | - | - | - | - | - | - | - |  |  |  |  |  |  |  |  |  |  |
| Ocular nasal discharge | |  | - | - | - | - | - | - | - | - | - |  |  |  |  |  |  |  |  |  |  |
| Eye redness | |  | - | - | - | - | - | - | - | - | - |  |  |  |  |  |  |  |  |  |  |
| Head swollen | |  | - | - | - | - | - | - | - | - | - |  |  |  |  |  |  |  |  |  |  |
| Ruffled feathers | |  | - | Y | Y | - | - | - | - | - | - |  |  |  |  |  |  |  |  |  |  |
| Reluctant to move | |  | - | - | - | - | - | - | - | - | - |  |  |  |  |  |  |  |  |  |  |
| Shedding | Buccal |  | - | Y | Y | Y | Y | Y | Y | Y | - |  |  |  |  |  |  |  |  |  |  |
|  | Cloacal |  | - | Y | Y | Y | Y | Y | - | - | - |  |  |  |  |  |  |  |  |  |  |
| General Sickness | | J7 | - | - | - | - | - | - | - | - | - |  |  |  |  |  |  |  |  |  |  |
| Sneezing | |  | - | - | - | - | - | - | - | - | - |  |  |  |  |  |  |  |  |  |  |
| Respiratory sound (rales) | |  | - | - | - | - | - | - | - | - | - |  |  |  |  |  |  |  |  |  |  |
| Ocular nasal discharge | |  | - | - | - | - | - | - | - | - | - |  |  |  |  |  |  |  |  |  |  |
| Eye redness | |  | - | - | - | - | - | - | - | - | - |  |  |  |  |  |  |  |  |  |  |
| Head swollen | |  | - | - | - | - | - | - | - | - | - |  |  |  |  |  |  |  |  |  |  |
| Ruffled feathers | |  | - | - | - | - | - | - | - | - | - |  |  |  |  |  |  |  |  |  |  |
| Reluctant to move | |  | - | - | - | - | - | - | - | - | - |  |  |  |  |  |  |  |  |  |  |
| Shedding | Buccal |  | Y | Y | Y | Y | Y | Y | Y | Y | Y |  |  |  |  |  |  |  |  |  |  |
|  | Cloacal |  | - | Y | Y | Y | Y | Y | - | - | - |  |  |  |  |  |  |  |  |  |  |
| General Sickness | | J8 | Y | Y | Y | - | - | - | - | - | - |  |  |  |  |  |  |  |  |  |  |
| Sneezing | |  | - | Y | Y | - | - | - | - | - | - |  |  |  |  |  |  |  |  |  |  |
| Respiratory sound (rales) | |  | - | - | - | - | - | - | - | - | - |  |  |  |  |  |  |  |  |  |  |
| Ocular nasal discharge | |  | - | - | - | - | - | - | - | - | - |  |  |  |  |  |  |  |  |  |  |
| Eye redness | |  | - | - | - | - | - | - | - | - | - |  |  |  |  |  |  |  |  |  |  |
| Head swollen | |  | - | - | - | - | - | - | - | - | - |  |  |  |  |  |  |  |  |  |  |
| Ruffled feathers | |  | - | Y | Y | - | - | - | - | - | - |  |  |  |  |  |  |  |  |  |  |
| Reluctant to move | |  | - | - | - | - | - | - | - | - | - |  |  |  |  |  |  |  |  |  |  |
| Shedding | Buccal |  | Y | Y | Y | Y | Y | Y | Y | Y | - |  |  |  |  |  |  |  |  |  |  |
|  | Cloacal |  | - | Y | Y | Y | Y | - | - | - | - |  |  |  |  |  |  |  |  |  |  |
| General Sickness | | J9 | - | Y | Y | - | - | - | - | - | - |  |  |  |  |  |  |  |  |  |  |
| Sneezing | |  | - | Y | Y | - | - | - | - | - | - |  |  |  |  |  |  |  |  |  |  |
| Respiratory sound (rales) | |  | - | - | - | - | - | - | - | - | - |  |  |  |  |  |  |  |  |  |  |
| Ocular nasal discharge | |  | - | - | - | - | - | - | - | - | - |  |  |  |  |  |  |  |  |  |  |
| Eye redness | |  | - | - | - | - | - | - | - | - | - |  |  |  |  |  |  |  |  |  |  |
| Head swollen | |  | - | - | - | - | - | - | - | - | - |  |  |  |  |  |  |  |  |  |  |
| Ruffled feathers | |  | - | Y | Y | - | - | - | - | - | - |  |  |  |  |  |  |  |  |  |  |
| Reluctant to move | |  | - | - | - | - | - | - | - | - | - |  |  |  |  |  |  |  |  |  |  |
| Shedding | Buccal |  | Y | Y | Y | Y | Y | Y | Y | Y | - |  |  |  |  |  |  |  |  |  |  |
|  | Cloacal |  | - | Y | Y | Y | - | - | - | - | - |  |  |  |  |  |  |  |  |  |  |
| General Sickness | | J10 | Y | Y | Y | - | - | - | - | - | - |  |  |  |  |  |  |  |  |  |  |
| Sneezing | |  | - | Y | Y | - | - | - | - | - | - |  |  |  |  |  |  |  |  |  |  |
| Respiratory sound (rales) | |  | - | - | - | - | - | - | - | - | - |  |  |  |  |  |  |  |  |  |  |
| Ocular nasal discharge | |  | - | - | - | - | - | - | - | - | - |  |  |  |  |  |  |  |  |  |  |
| Eye redness | |  | - | - | - | - | - | - | - | - | - |  |  |  |  |  |  |  |  |  |  |
| Head swollen | |  | - | - | - | - | - | - | - | - | - |  |  |  |  |  |  |  |  |  |  |
| Ruffled feathers | |  | - | Y | Y | - | - | - | - | - | - |  |  |  |  |  |  |  |  |  |  |
| Reluctant to move | |  | - | - | - | - | - | - | - | - | - |  |  |  |  |  |  |  |  |  |  |
| Shedding | Buccal |  | Y | Y | Y | Y | Y | Y | Y | Y | - |  |  |  |  |  |  |  |  |  |  |
|  | Cloacal |  | - | Y | Y | Y | Y | - | - | - | - |  |  |  |  |  |  |  |  |  |  |
