# Supplementary material for: Infectivity and transmissibility of H9N2 avian influenza virus in chickens and wild terrestrial birds
Source: Vet Res. 2013 Oct 17;44(1):100. doi: 10.1186/1297-9716-44-100 (PMC4015117; doi:10.1186/1297-9716-44-100)
Supplement: Additional file 7 — Mean viral titers in buccal and cloacal swabs taken from the infected groups on 3 dpi and from contact groups on 4 dpi. Viral titres are presented as log10 EID50/mL determined in EHE inoculated with buccal and cloacal swabs taken from the infected groups on 3 dpi and from the contact groups on 4 dpi. SD, denotes standard deviation. [file 1297-9716-44-100-S7.docx]

| **Species** | **Infection route** | **Day post**  **infection** | **Buccal swabs** | | | **Cloacal swabs** | | |
| --- | --- | --- | --- | --- | --- | --- | --- | --- |
|  |  |  | Median | Mean | SD | Median | Mean | SD |
| Broilers | infected | 3 | 3.45 | 3.57 | 0.47 | 2.90 | 2.96 | 0.42 |
| Sparrows | contact | 4 | 1.85 | 2.00 | 0.50 | 2.90 | 2.66 | 0.77 |
| Sparrows | infected | 3 | 2.18 | 2.15 | 0.35 | 3.40 | 3.50 | 0.40 |
| Broilers | contact | 4 | 3.15 | 3.00 | 0.49 | 2.60 | 2.70 | 0.24 |
| Quail | infected | 3 | 7.4 | 7.49 | 0.78 | 4.99 | 5.17 | 2.19 |
